# HBV Subgenotypes D1, D2, D-del! Are ‘Old‘ Genotyping Methods Interpreted Correctly?

**DOI:** 10.5812/hepatmon.13048

**Published:** 2013-07-01

**Authors:** Samad Amini-Bavil-Olyaee, Frank Tacke, Seyed Moayed Alavian

**Affiliations:** 1 Department of Molecular Microbiology and Immunology, Keck School of Medicine, University of Southern California, Harlyne J. Norris Cancer Research Tower, Los Angeles, CA 90033, USA; 2 Department of Medicine III, RWTH-University Hospital Aachen, Aachen, Germany; 3 Baqiyatallah Research Center for Gastroenterology and Liver Diseases, Baqiyatallah University of Medical Sciences, Tehran, IR Iran

**Keywords:** Hepatitis B virus, Genotype

Infections with the hepatitis B virus (HBV) are one of the major global public health problems. HBV has been categorized into different genotypes and subgenotypes that are distributed distinctively around the world. These classifications provide important information as the genotypes differ with respect to the clinical course of disease as well as in their response to antiviral therapy, and subgenotyping allows relevant conclusions about transmission routes, global or local spreading of infections or phylogenetic relations between viral strains (1). Several molecular virology methods are utilized to (sub) genotype HBV including direct sequencing (as gold standard), restriction fragment length polymorphism (RFLP), restriction fragment mass polymorphism (RFMP), oligonucleotide microarray chip (DNA Chip), INNO-LiPA, and a range of (real-time)-PCRs with distinct advantages and limitations (2). Among these methods for (sub) genotyping, RFLP is widely considered as one of the most favorable methods among scientists, since it is technically simple, robust, inexpensive and can be established in virtually any laboratory with basic molecular biology facilities. As a consequence, RFLP has been frequently and successfully employed for many studies worldwide including large cohorts of HBV-infected patients (3-5). Despite its clear benefits, this assay also has a number of pitfalls that may impede to assess the correct HBV genotype (4). For instance, any variation within HBV genome that affects the assay’s enzyme restriction site(s) negatively impacts the outcome of the method. HBV genomic variations regularly occur in patients due to natural viral evolution or endogenous and/or exogenous pressures such as immune responses, antiviral therapeutic regimes and/or vaccination (6). However, the mal-interpretation of results obtained with the RFLP assay is also an important trap that needs to be considered by researchers using this assay. In this editorial, we would like to illustrate our concern by commenting on several studies applying an established RFLP-based HBV genotyping method, in which the results were (to our opinion) interpreted incorrectly (Table 1) (7-12). 

**Table 1.** Epidemiological studies on HBV Genotyping and Subgenotyping with misinterpretations of the RFLP method introduced by Lindh et al

First Author	Incorrect Interpretation	Correct Interpretation	Geographical Region	Year	Published in	Reference
**Kaklikkaya N**	subgenotypes D2, D-del and D1	genotype D	Turkey	2012	Saudi Med J	(7)
**Neisi N**	subgenotypes D2 and B6	genotype D	Iran	2011	Jundishapur J Microbiol	(10)
**Kumar A**	subgenotypes A1, A2, D2 and D3	genotypes A and D	India	2011	Indian J Virol	(8)
**Sunbul M**	subgenotypes D2 D-del, D1 and D3	genotype D	Turkey	2005	World J Gastroenterol	(11)
**Leblebicioglu H**	subgenotypes D2, D-del and D1	genotype D	Turkey	2004	Clin Microbiol Infect	(9)
**Theamboonlers A**	subgenotypes C1, C7, C8 and B1	genotypes C and B	Thailand	1999	Ann Trop Med Parasitol	(12)

The latest of these studies (published in 2012) investigated HBV genotypes and subgenotypes among patients living in the Eastern Black Sea region of Turkey (7). The authors reported that HBV genotype D is most prevalent in Turkish patients. Indeed, their findings are fully in line with the current data regarding HBV molecular epidemiology in this region, where genotype D and its subgenotype D1 of HBV is considered most prevalent. Surprisingly, in this study, HBV subgenotype D2 (n = 122, 97.6%) was reported as the most frequent HBV subgenotype followed by the subgenotypes D1 and D-del. However, we believe that this specific conclusion on the subgenotypes is the result of a misinterpretation of the particular RFLP methodology utilized in this study. This investigation, as well as the other studies listed in Table 1, employed a PCR-RFLP based method that was originally introduced by Lindh and coworkers in 1998, when no universal taxonomy classification for HBV “subgenotype” existed yet (13). Importantly, the first standard classification of HBV “subgenotypes” was established based on phylogenetic analyses of HBV full genome sequences in 2004 (14). The genotyping method developed by Lindh et al is a RFLP based-method and relies on digestion analysis of the HBV pre-S gene, a highly conserved region in HBV genome. Lindh et al employed the previously defined HBV genotypes (A to F) and developed their RFLP assay, which was only able (and intended!) to determine HBV “genotypes”, but not “subgenotypes”. Accordingly, Lindh at al introduced several RFLP migration patterns of the digested segments of pre-S amplicon and then named A1-A3 (corresponding to genotype A), B1-B3 (corresponding to genotype B), C1-C6 (corresponding to genotype C), D1-D2 and D-del [due to a deletion in the pre-S region] (all corresponding to genotype D), E1-E2 (corresponding to genotype E), and F1-F2 (corresponding to genotype F) (13). Importantly, the various electrophoresis migration patterns corresponded to a solitary “genotype” of HBV. Quite accidentally, these abbreviated names are strikingly similar to the current nomenclature of HBV “subgenotypes” abbreviations (A1-A4, B1-B8, C1-C10, D1-D7, E, F1-F4, G and H), which are defined by sequence divergence of between 4%-8% within the HBV full-length genome sequence (15). In fact, the designated names of the electrophoresis migration patterns in Lindh’s technique have absolutely no association with the modern HBV “subgenotyping” categorization and its nomination! The very similar abbreviations between Lindh’s RFLP method and the official subgenotype classification have thus misled several research groups around the world (Table 1), resulting in misinterpretations on the prevalence of HBV subgenotypes in distinct regions of the world. Thus, we would like to strongly encourage scientists to apply state-of-the-art methods for their HBV molecular epidemiology studies such as full-length genome sequencing followed by further evolutionary analysis for the important HBV subgenotype classifications. When using ‘older’ methods such as RFLP analysis, the limitations of the method and the historical designation of similar terms to electrophoresis migration patterns need to be considered to avoid incorrect interpretations. Also, the scientific reviewers of our community need to be aware of the details of the virological methods to preclude publication of inaccurate results. 
